# COPD and disease-specific health status in a working population

**DOI:** 10.1186/1465-9921-14-61

**Published:** 2013-06-02

**Authors:** Koichi Nishimura, Satoshi Mitsuma, Atsuko Kobayashi, Mikako Yanagida, Kazuhito Nakayasu, Yoshinori Hasegawa, Paul W Jones

**Affiliations:** 1 Department of Pulmonary Medicine, National Center for Geriatrics and Gerontology, 35 Gengo, Morioka, Obu, Aichi 474-8511, Japan; 2 Niigata Association of Occupational Health Incorporated, Niigata, Japan; 3 Data Research Section, Kondo P.P. Inc, Osaka, Japan; 4 Division of Respiratory Medicine, Department of Medicine, Nagoya University Graduate School of Medicine, Nagoya, Japan; 5 Division of Clinical Science, St. George’s Hospital Medical School, London, UK

**Keywords:** Chronic obstructive pulmonary disease, St. George’s Respiratory Questionnaire, Symptoms and COPD, Smoking and health, Health related quality of life

## Abstract

**Background:**

It has been debated whether treatment should be started early in subjects with mild to moderate COPD. An impaired health status score was associated with a higher probability of being diagnosed with COPD as compared with undiagnosed COPD.

**Purpose:**

To investigate the health status in a healthy working population, to determine reference scores for healthy non-smoking subjects, and to investigate the relationship between their health status and airflow limitation.

**Methods:**

A total of 1333 healthy industrial workers aged ≥40 years performed spirometry and completed the St. George’s Respiratory Questionnaire (SGRQ) and the COPD Assessment Test (CAT).

**Results:**

The prevalence of COPD defined by the fixed ratio of the forced expiratory volume in one second (FEV_1_)/forced vital capacity (FVC) was 10.9%, and the prevalence defined by the Lower Limit of Normal was 5.0%. All SGRQ and CAT scores were skewed to the milder end. In 512 non-smoking subjects with normal spirometry, the mean SGRQ score was 5.7, and the mean CAT score was 5.8. In 145 people with COPD defined by the fixed ratio, the mean SGRQ score was 7.9, with a zero score in 6.9% of the subjects. Using the CAT, the mean score was 7.3, with 7.6% of the scores being zero. The scores in patients identified using the Lower Limit of Normal approach were: SGRQ 8.4 (13.4% had a score of zero) and CAT 7.4 (13.4% had a score of zero). Although the 95th percentiles of the Total, Symptoms, Activity, and Impact scores of the SGRQ and CAT sores were 13.8, 34.0, 23.4, 7.2 and 13.6 in the 512 healthy non-smoking subjects, respectively, they were also distributed under their upper limits in over 80% of the COPD subjects.

**Conclusion:**

The COPD-specific health status scores in a working population were good, even in those with spirometrically diagnosed COPD. All scores were widely distributed in both healthy non-smoking subjects and in subjects with COPD, and the score distribution overlapped remarkably between these two groups. This suggests that symptom-based methods are not suitable screening tools in a healthy general population.

## Background

It has been reported that chronic obstructive pulmonary disease (COPD) remains under-diagnosed, with the diagnosis being commonly missed or delayed until the disease is advanced. Although some investigators have proposed targeted, systematic case-identification as a strategy to reduce the burden of COPD [1,2], others have recommended that spirometry should not be used to screen for airflow obstruction in asymptomatic individuals, since little evidence exists to support the benefit of interventions, except for smoking cessation, in early disease [3,4]. Furthermore, it has been debated whether treatment should be started early in subjects with mild to moderate COPD [5].

Several epidemiological studies have shown that only a small proportion of subjects with COPD have had a prior diagnosis, and that subjects with unrecognized COPD usually have mild to moderate airflow limitation [6-9]. Much less information is available about the health of patients identified in this way. The Epidemiologic Study of COPD in Spain (EPI-SCAN) found that 27% of identified COPD cases had a previous diagnosis of COPD, and that an impaired health status score was associated with a higher probability of being diagnosed with COPD as compared with undiagnosed COPD [7].

We hypothesized that health status measurements may identify undiagnosed subjects with mild to moderate COPD. The purpose of the present study was to investigate the health status in a healthy working population, to determine reference scores for healthy non-smoking subjects, and to investigate the relationship between their health status and airflow limitation. The health status was assessed by the St. George’s Respiratory Questionnaire (SGRQ) and the COPD Assessment Test (CAT™) [10-12]. Two definitions of airflow limitation were used: a fixed ratio of the FEV_1_/FVC < 0.7, and the Lower Limit of Normal (LLN) definition [13,14].

## Materials and methods

### Subjects

The study was conducted between September 2010 and March 2011 at the Niigata Association of Occupational Health Incorporated, Niigata, Japan. The study subjects were healthy industrial workers over forty years-old who underwent annual health checks at this Association. All had a comprehensive health screening, including conventional spirometry, as well as a chest radiograph. The exclusion criteria for this analysis included: 1) abnormal findings of the pulmonary parenchyma and chest wall revealed on chest radiographs; 2) receiving a thoracotomy in the past; 3) any admission to a hospital during the preceding three months; 4) any physician-diagnosed pulmonary diseases including lung cancer, pulmonary tuberculosis, bronchiectasis and non-tuberculous mycobacteriosis; 5) a history of cancer or malignant disorders; and 6) unstable complications of cardiovascular, neuromuscular, renal, endocrinological, hematological, gastrointestinal, and hepatic co-morbidities. Written informed consent was obtained from all participants. The present study was approved by the ethics committee of the Niigata Association of Occupational Health Incorporated.

In this study, COPD was spirometrically defined as subjects with an airflow limitation of FEV_1_/FVC less than a fixed ratio, 0.7, or a LLN without bronchodilator administration. Healthy subjects were defined as a FEV_1_ of >85% pred or a FEV_1_/FVC of >0.7, forming two groups: subjects with a smoking history of ≥10 pack-yrs, and non-smoking subjects with a smoking history of < 1 pack-yr. This definition was similar to that of the Evaluation of COPD Longitudinally to Identify Predictive Surrogate End-points (ECLIPSE) study [15].

### Methods

All eligible subjects finished the following examinations on the same day. Spirometry was performed with the use of nose clips in the sitting position with a Spiro Sift sp-470™ Spirometer (Fukuda Denshi Co., Ltd., Tokyo, Japan). All measurements were performed by a laboratory technician (A.K.) in accordance with guidelines published by the American Thoracic Society and the European Respiratory Society [16]. The spirometric FVC and FEV_1_ values were the largest FVC and largest FEV_1_ selected from data obtained from at least three acceptable forced expiratory curves, even if these values were not obtained from the same curve [17]. The predicted values for pulmonary function were calculated based on the proposal from the Japanese Respiratory Society [18]. The LLN for the Japanese population was calculated using the method described by Osaka et al. [19]. The Japanese versions of the SGRQ (version 2) and CAT were administered under supervision prior to the pulmonary function tests. These Japanese versions have been previously validated [20,21]. The scores for three components (Symptoms, Activity, and Impact) and the Total score of the SGRQ were then calculated, as well as the CAT score. The participants also answered additional questions to investigate their smoking status and history. Information about their radiographic findings was obtained from annual health examinations.

### Statistics

All results are expressed as means ± SD. Any missed SGRQ items were handled according to the developers’ instructions in the SGRQ manual. A Mann–Whitney *U*-test was used to compare the CAT and SGRQ scores between two groups. A p value of less than 0.05 was considered to be statistically significant. Monte Carlo and bootstrap methods with 1,000 bootstrap reps were used to calculate the 95th percentile of the scores in healthy, non-smoking subjects [22].

## Results

A total of 1382 subjects participated at the beginning of the present study. However, 49 subjects were excluded from the data analysis because of uncertainty in their smoking history or one of the exclusion criteria. Therefore, a total of 1333 consecutive subjects (871 males) were analysed. The average age of the subjects was 56.0 years. The mean FEV_1_/FVC ratio was 78.4%, range 40.2% to 94.4%. Their demographic details are shown in Table 1.

**Table 1:** Demographic details and spirometric results from all 1333 subjects

	**Total subjects **	**Age**	**Male **	**Cumulative smoking**	**Never smoker **	**Prior diagnosis**		**FEV**_**1**_	**FEV**_**1**_**/FVC**
						**Number (%)**			
	**number**	**Years**	**number (%)**	**pack-years**	**number (%)**	**Asthma**	**COPD**	**% pred**	**%**
All subjects	1333	56.0 ± 8.2	871 (65.3%)	17.0 ± 21.7	556 (41.7%)	21 (1.6%)	7 (0.5%)	95.8 ± 14.8	78.4 ± 6.7
Non-COPD defined by fixed ratio	1188	55.4 ± 8.1	731 (61.5%)	14.3 ± 19.3	539 (45.4%)	18 (1.5%)	0 (0%)	98.2 ± 13.0	80.1 ± 4.5
COPD defined by fixed ratio	145	61.0 ± 7.7	140 (96.6%)	38.4 ± 27.5	17 (11.7%)	3 (2.1%)	7 (4.8%)	76.6 ± 15.1	64.6 ± 5.6
Non-COPD defined by LLN	1266	55.7 ± 8.2	807 (63.7%)	15.6 ± 20.1	548 (43.3%)	19 (1.5%)	1 (0.1%)	97.3 ± 13.5	79.4 ± 5.2
COPD defined by LLN	67	60.6 ± 7.2	64 (95.5%)	43.0 ± 32.5	8 (11.9%)	2 (3.0%)	6 (8.9%)	68.8 ± 13.0	60.3 ± 5.7
Healthy non-smoking subjects¶#	512	56.2 ± 8.2	123 (24%)	0.0 ± 0.1	495 (96.7%)	8 (1.6%)	0 (0%)	104.5 ± 11.3	81.4 ± 4.4
Healthy smoking subjects¶*	399	54.7 ± 8.0	373 (93.5%)	29.1 ± 15.9	0 (0%)	6 (1.5%)	0 (0%)	98.0 ± 9.0	79.7 ± 4.1
COPD/GOLD stageI	60	60.8 ± 7.5	57 (95.0%)	33.4 ± 23.3	10 (16.7%)	1 (1.7%)	0 (0%)	90.4 ± 8.8	67.4 ± 2.6
COPD/GOLD stageII	79	61.4 ± 8.0	78 (98.7%)	42.1 ± 30.2	6 (7.6%)	1 (1.3%)	7 (8.9%)	68.6 ± 7.7	63.6 ± 4.8

¶ FEV_1_ of >85% pred and FEV_1_/FVC of >0.7, # smoking history of < 1 pack-yr, * smoking history of ≥10 pack-yrs.

Using the fixed ratio of the FEV_1_/FVC < 0.7, 145 subjects were diagnosed with COPD, which included 140 out of 871 males (16.1%) and 5 out of 462 females (1.1%). Using the Global Initiative for Chronic Obstructive Lung Disease (GOLD) criteria [23], 41.3% were in GOLD 1, 54.5% in GOLD 2 and 4.1% in GOLD 3 & 4, (Table 1). Out of the 1188 subjects without COPD, there were 512 nonsmoking subjects, 399 smoking subjects and 277 others who smoked between 1 and 10 pack years. Using the LLN definition, 67 subjects had COPD, which included 64 males (7.3%) and 3 females (0.1%), but 1266 were not considered to have COPD. The overall prevalence of COPD was 10.9% defined by the fixed ratio, and 5.0% by the LLN. The greater prevalence in males than in females was associated with the much higher smoking rate in men.

The distributions of the Total score of the SGRQ and the CAT score are shown in Table 2, and the three components of the SGRQ are shown in Table 3. Since all of the scores were skewed to the milder ends, a floor effect was seen in all of the scores, most marked with the Impact score of the SGRQ. The Total, Symptoms, and Impact scores of the SGRQ were significantly different between subjects with and without COPD as defined by the fixed ratio (p = 0.040, <0.001 and 0.001, respectively, Mann–Whitney *U*-test), but the SGRQ Activity score and the CAT score were not different between the two groups. The SGRQ Symptoms and Impact scores were significantly higher in subjects with COPD as defined by the LLN than in subjects without COPD (both p < 0.001), but the SGRQ Total and Activity scores and the CAT score were not significantly different between the two groups.

**Table 2:** Score distribution of the Total score of the SGRQ and CAT scores

	**SGRQ Total (0–100)**					**CAT (0–40)**				
	**Mean**	**Median**	**SD**	**Max**	**Floor effect**	**Mean**	**Median**	**SD**	**Max**	**Floor effect**
All subjects	6.4	5.3	5.7	51.5	8.9%	6.4	6.0	4.6	26.0	7.4%
Non-COPD defined by fixed ratio	6.3^1)^	5.3	5.4	43.2	9.1%	6.3	6.0	4.5	24.0	7.3%
COPD defined by fixed ratio	7.9	6.4	7.6	51.5	6.9%	7.3	6.0	5.2	26.0	7.6%
Non-COPD defined by LLN	6.3	5.3	5.6	51.5	8.6%	6.4	6.0	4.5	24.0	7.0%
COPD defined by LLN	8.4	6.7	7.5	34.0	13.4%	7.4	6.0	5.9	26.0	13.4%
Healthy non-smoking subjects¶#	5.7^2)3)4)5)^	4.9	4.9	43.2	10.5%	5.8^2)3)4)^	5.0	4.4	23.0	9.4%
Healthy smoking subjects¶*	6.8	5.8	5.5	37.1	7.5%	6.8	6.0	4.4	24.0	5.3%
COPD/GOLD stageI	8.1	5.7	9.2	51.5	5.0%	7.2	6.0	5.1	21.0	6.7%
COPD/GOLD stageII	7.5	6.4	6.1	26.8	8.9%	7.2	6.0	5.3	26.0	8.9%

¶ FEV_1_ of >85% pred and FEV1/FVC of >0.7, # smoking history of <1 pack-yr, * smoking history of ≥10 pack-yrs.

1) p < 0.05 compared with COPD defined by fixed ratio.

2) p < 0.01 compared with healthy smoking subjects.

3) p < 0.01 compared with COPD defined by fixed ratio.

4) p < 0.05 compared with COPD/GOLD stageII.

5) p < 0.05 compared with COPD defined by LLN.

**Table 3:** Score distribution of the three components of the SGRQ

	**SGRQ Symptoms (0–100)**					**SGRQ Activity (0–100)**					**SGRQ Impact (0–100)**				
	**Mean**	**Median**	**SD**	**Max**	**Floor effect**	**Mean**	**Median**	**SD**	**Max**	**Floor effect**	**Mean**	**Median**	**SD**	**Max**	**Floor effect**
All subjects	15.8	13.5	14.0	81.8	19.1%	9.1	6.0	9.1	59.5	34.0%	1.8	0.0	4.9	47.2	77.8%
Non-COPD defined by fixed ratio	15.0^1)2)3)^	12.9	13.5	74.0	20.1%	9.1	6.0	8.9	59.5	33.3%	1.7^1)3)^	0.0	4.7	44.3	79.0%
COPD defined by fixed ratio	21.7	18.8	16.3	81.8	10.3%	9.3	6.0	10.9	48.9	39.3%	2.7	0.0	6.0	47.2	67.6%
Non-COPD defined by LLN	15.4^4)^	12.9	13.7	81.8	19.1%	9.1	6.0	8.9	59.5	33.4%	1.7^4)^	0.0	4.8	47.2	78.8%
COPD defined by LLN	22.0	18.8	16.5	76.3	17.9%	9.5	6.0	11.9	43.0	44.8%	3.4	0.0	5.6	26.6	59.7%
Healthy non-smoking subjects¶#	12.5^1)2)3)4)^	11.0	11.7	74.0	23.8%	8.9	6.2	9.0	59.5	36.1%	1.4^1)3)4)8)^	0.0	4.7	44.3	82.2%
Healthy smoking subjects¶*	17.3^1)4)7)^	15.4	14.0	72.2	16.0%	9.1	6.0	8.3	45.5	28.8%	1.9^4)9)^	0.0	5.0	36.3	75.9%
COPD/GOLD stageI	21.9	20.3	17.6	81.8	6.7%	9.2	6.0	11.3	48.9	38.3%	3.1	0.0	7.9	47.2	73.3%
COPD/GOLD stageII	21.3	18.7	15.8	76.3	13.9%	8.8	6.0	10.1	43.0	40.5%	2.3	0.0	4.2	18.1	67.1%

¶ FEV_1_ of >85% pred and FEV_1_/FVC of >0.7, # a smoking history of <1 pack-yr, * a smoking history of ≥10 pack-yrs.

1) p < 0.01 compared with COPD defined by fixed ratio.

2) p < 0.01 compared with COPD/GOLD stageI.

3) p < 0.01 compared with COPD/GOLD stageII.

4) p < 0.01 compared with COPD defined by LLN.

5) p < 0.01 compared with healthy smoking subjects.

6) p < 0.05 compared with COPD/GOLD stageII.

7) p < 0.05 compared with COPD defined by LLN.

8) p < 0.05 compared with healthy smoking subjects.

9) p < 0.05 compared with COPD defined by fixed ratio.

**Figure 1:**
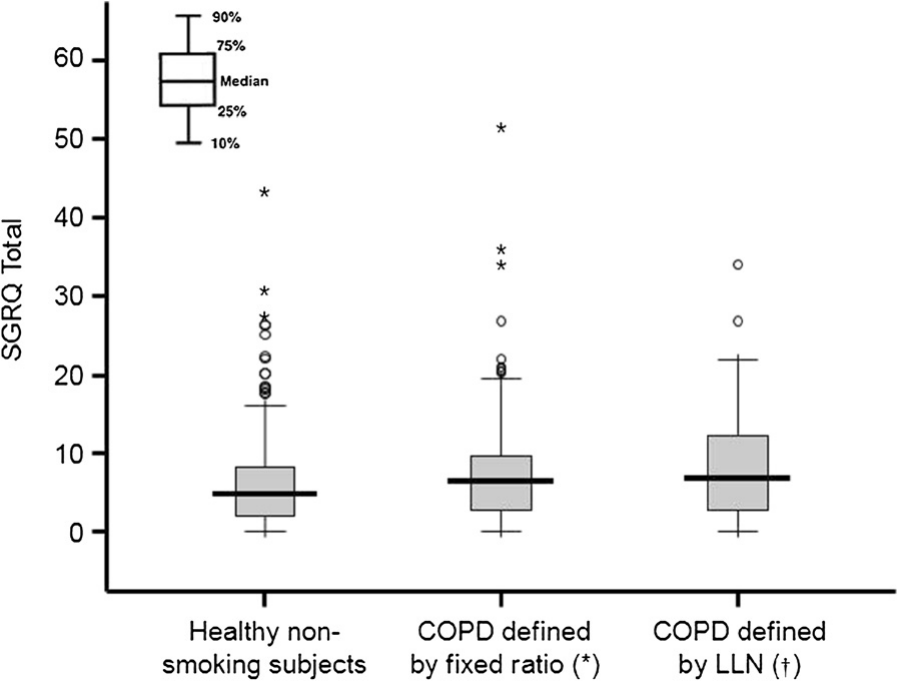
**Box plots representing the score distributions of the SGRQ Total score in healthy non-smoking subjects, COPD defined by the fixed ratio and COPD defined by the LLN.** * p < 0.01, †p < 0.05; both significant differences in the scores were observed for healthy non-smoking subjects.

**Figure 2:**
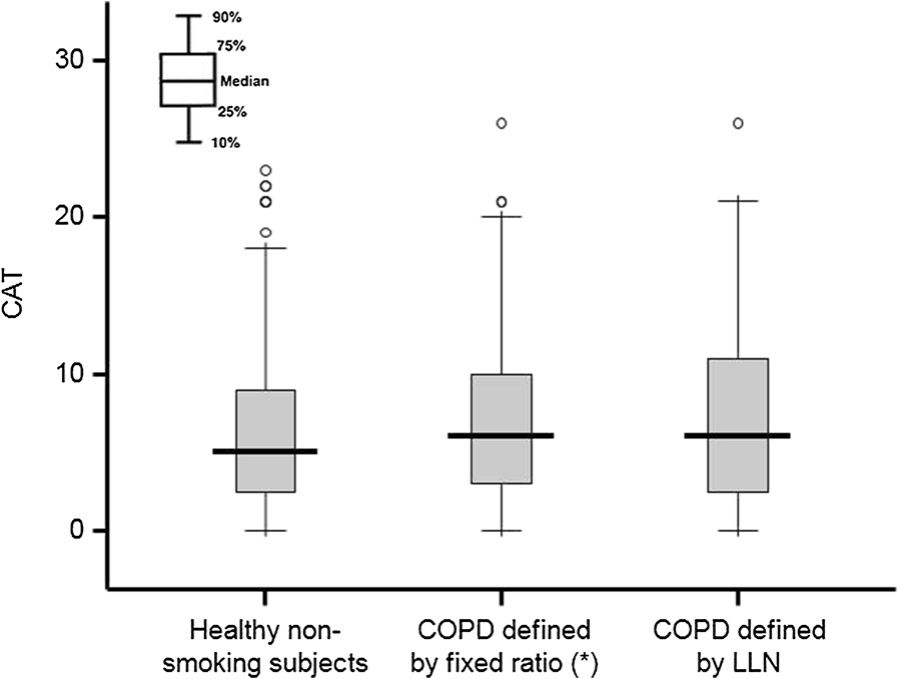
**Box plots representing the score distributions of the CAT score in healthy non-smoking subjects, COPD defined by the fixed ratio and COPD defined by the LLN.** * p < 0.01; significant differences in the scores were observed for healthy non-smoking subjects.

As a secondary endpoint, it was an objective to determine reference values for each score. Therefore, we calculated the 95th percentile of the scores in 512 healthy, non-smoking subjects using the Monte Carlo method and used this as the upper limit of normal. For the SGRQ, these values were a SGRQ Total score 13.8, Symptoms score 34.0, Activity score 23.4, and Impact score 7.2; for the CAT, it was 13.6 (which rounds to 14, since the CAT score does not contain decimals). In the subjects with COPD, over 80% of the SGRQ and CAT scores fell below these values. The median SGRQ Symptoms score in the healthy subjects was 11.0, and in those subjects diagnosed with COPD using the fixed ratio, 24.1% had scores less than this. Thus, the score distributions overlapped widely between healthy, non-smoking subjects and those identified by spirometry, regardless of the definition used (Figures 1 and 2).

## Discussion

This study showed that the health status scores of undiagnosed COPD patients in a working population were very close to the scores in subjects without COPD from the same study population, regardless of the health status score used or the spirometric method used to diagnose COPD. This supports a previous observation that people with undiagnosed COPD and identified on spirometric screening have better SGRQ scores than those previously diagnosed [7]. Three main conclusions may be drawn from this. First, undiagnosed people with COPD who are in paid employment have a well preserved health status. Second, in such populations, symptom-based screening tools are unlikely to identify undiagnosed patients, or act as case-finding tools. Third, setting aside smoking cessation (which ought to be a universal health care aim) and the identification of patients with a high frequency of exacerbations but minimal symptoms (Group C using the GOLD 2011 classification) [23], this study lends little support to screening studies in healthy populations.

The participants in this study were healthy industrial workers over 40 years-old in Japan. The prevalence of COPD defined by the fixed ratio was 10.9%, whereas that defined by the LLN was 5.0%. Although there have been only a few population-based surveys regarding the prevalence of COPD in Japan, these figures are very similar to those reported in the Nippon COPD Epidemiology (NICE), in which the prevalence defined by the fixed ratio was 10.9% [6]. Similarly, in a community-based annual health check, Osaka et al. also reported that 10.6% had COPD as defined by the fixed ratio and 6.4% had COPD as defined by the LLN [19]. Thus, the prevalence of COPD is similar to previous studies in Japan.

Although many population-based surveys using spirometry have reported the under-diagnosis of COPD, this frequency is highly variable. Analyses of population-based data from the US suggest that 63.3% had no prior or current reported diagnosis of any obstructive lung disease [24]. This percentage ranges from 19 to 27% in European studies, but is the lowest in Latin America (11.3%) and in Japan (9.4%) [6,25]. Therefore, the small number of subjects in our study with a prior diagnosis of COPD may also be related to their national background.

To the best of our knowledge, there has been no definition of “asymptomatic” COPD. A simple answer to the choice of yes or no for a single question may not be reliable or valid to determine the presence of symptoms. Nonetheless, a reproducible and quantitative measurement of symptoms is necessary. Since symptoms are one of the essential components of health status, some subjective markers obtained from established methods of patient-reported outcomes could be candidates. It is reasonable to argue that subjects with a floor effect (zero score) on their scores obtained from disease-specific instruments are asymptomatic for that disease. In our study, approximately 7% of the subjects with COPD had zero scores whether using the SGRQ Total score or the CAT, and hence we conclude that only a small number of subjects with undiagnosed COPD are free of any symptoms. Nonetheless, people with asymptomatic COPD may have some problems with their health, for example comorbidity.

Although the Total score of the SGRQ and the CAT score were significantly better in healthy, non-smoking subjects than in subjects with COPD, the difference in the scores is very small, and the score distributions were also similar. All of the scores were widely distributed in both groups, and the score distribution overlapped to a large extent between the two groups. Although we have created reference values for healthy, non-smoking subjects, it became apparent that they are not useful to identify undiagnosed people with COPD.

Ohar et al. studied 3955 subjects using work-related medical evaluations, and observed that respiratory symptoms were reported by 92% of smokers with airflow limitation, 76% of smokers with normal spirometry, and 73% of non-smokers, and concluded that the high prevalence of symptoms resulted in a poor predictive value for COPD [26]. However, they examined symptoms using a simple binary choice of yes or no during face-to-face interviews. Although it is difficult to directly compare their results with ours due to the differences in methodology, their conclusion is similar to ours. In the ECLIPSE study, Agusti et al. reported SGRQ scores of 9.6 in non-COPD smokers and 4.5 in non-smoking controls. These estimates are also quite similar to ours. However, the scores in their patients (diagnosed with COPD and under hospital outpatient treatment) were much higher, with a mean of 50.1 [27]. This large difference between the studies is compatible with the observation that SGRQ scores in undiagnosed COPD patients identified by spirometric screening were considerably better than patients with a prior diagnosis of COPD [7]. The clear inference to be drawn is that COPD patients are diagnosed because of many noticeable symptoms, and it is reasonable to draw the opposite conclusion, that many of those who are not diagnosed have fewer symptoms. This means that in a general population, the use of symptomatic instruments may not be a sensitive method of identifying people who should undergo spirometric testing. This conclusion is supported by a report from Salameh et al. that the Diagnosis Score for COPD (DS-COPD) differentiated between COPD and other individuals with respiratory symptoms, but had no utility in identifying asymptomatic individuals [28].

Some limitations of the present study should be mentioned. Since the study subjects were not randomly sampled, there was a risk of sample bias. For example, healthy people may have been more likely to volunteer, although people with some symptoms may have also taken the opportunity to have a more thorough ‘check-up’. The study was limited by a relatively small number of participants; however, it is clear from the distribution in the SGRQ and CAT scores, that a great number of participants would have increased the precision of the mean estimates, but would not have materially altered the overall findings. In addition, GOLD defines airflow limitation as a post-bronchodilator FEV_1_/FVC < 0.70. However, this study did not evaluate post-bronchodilator values. Although the diagnosis of COPD may require somewhat more than only spirometry in clinical practice, the diagnosis was made only from spirometric information in the present study. The SGRQ and CAT are valid instruments for measuring health status in patients with diagnosed COPD, but most of the participants in our study were not patients with known COPD but rather healthy workers, and thus the SGRQ and CAT may have been inappropriate questionnaires for this study population. That having been said, most patients had scores that were not zero, indicating that most participants felt that some of the items did reflect their health state, even though they did not have a COPD diagnosis.

## Conclusion

Although the health status scores were statistically significantly different between healthy non-smoking subjects and those with COPD, the scores were widely distributed in both groups, and the score distribution overlapped widely. In a working population, a low level of respiratory-related symptom may be present in many subjects without airflow limitation, but there are also some apparently asymptomatic people with COPD (as judged by the SGRQ and CAT). We conclude that most working people who do meet the spirometric criteria for COPD are likely to have a well-preserved health status.

## Competing interests

The authors declare that they have no competing interests.

## Authors’ contributions

KN planned the study design, and was a major contributor in writing the manuscript. SM was the physician responsible for all participants. AK and MY participated in the data collection and the care for the participants. KN performed the statistical analysis. YH and PWJ contributed to the data analysis and interpretation and editing of the manuscript. All authors read and approved the final manuscript.
